# Sensory-Guided Identification and Characterization of Kokumi-Tasting Compounds in Green Tea (*Camellia sinensis* L.)

**DOI:** 10.3390/molecules27175677

**Published:** 2022-09-02

**Authors:** Jiachun Lu, Yanyan Cao, Yani Pan, Sifan Mei, Gang Zhang, Qiang Chu, Ping Chen

**Affiliations:** Tea Research Institute, Zhejiang University, 866 Yuhangtang Road, Hangzhou 310058, China

**Keywords:** *kokumi* peptides, sensory-guided, identification, characterization, recombination

## Abstract

The chemical substances responsible for the *kokumi* taste of green tea infusion are still unclear. Here, we isolated the *kokumi* compound-containing fractions from green tea infusion through ultrafiltration, and the major *kokumi* compounds were characterized as γ-Glu-Gln and γ-Glu-Cys-Gly (GSH) through ultra-high-performance liquid chromatography-tandem mass spectrometry (UHPLC-MS). The results indicated that peptides and amino acids were essential compounds in the kokumi-enriched fractions for conducting the sense of *kokumi*. L-theanine had an enhancing effect on the *kokumi* taste of green tea infusion, which was confirmed in the sensory reconstitution study. Thus, peptides, especially γ-Glu-Gln and GSH, are the major *kokumi* compounds in green tea infusion, which has the potential of improving the flavor of tea beverages.

## 1. Introduction

Certain foods have characteristics such as thickness, continuity, and mouthfulness, which is referred to as *kokumi*. *Kokumi* is a taste sensation quite different from the five basic taste attributes (sweet, salty, sour, bitter, and *umami*). Like other taste attributes, *kokumi* has its own receptor called the calcium-sensing receptor (CaSR) [1]. Although the aqueous solution of kokumi-tasting compounds barely has flavor, they substantially enhance the thickness, continuity, complexity, and mouthfulness of foods or beverages as added [2]. Besides, *kokumi* peptides may improve the sensory quality of reduced-fat products in terms of thick flavor, aftertaste, and oiliness [3]. They have the potential to improve the flavor of foods and beverages and have applications in diet foods [4].

Green tea is a widely consumed beverage, which has a delicate fragrance, rich and thick taste, and numerous health benefits [5]. Some green teas, without fermentation and maturing steps during processing, have strong intensity of *kokumi* taste. The compounds responsible for the *kokumi* taste in green tea infusion are still unclear.

A series of peptides have been reported to influence the *kokumi* taste of animal-derived, plant-based, and fermented food [4]. A group of γ-glutamyl peptides performed specific kokumi-promoting effects in parmesan cheese [6] and gouda cheese [7]. γ-Glu-Val-Gly is the key peptide contributing to *kokumi* and *umami* taste in fermented shrimp [8], scallops [9], and soy sauce [10]. For tea infusion, *kokumi* compounds may also be peptides, which need further verification.

A sensory-guided isolation technology can quickly locate the new taste activity compounds from an unknown mixture [11]. This method determines the intensity of the key flavor by taste dilution analysis (TDA) and taste dilution factor (TD-factor) for different substances. Substances with significant taste intensity are separated for further analysis. This strategy has been successfully applied to identify the critical taste compounds, such as the *umami* peptide of tempeh (Indonesian fermented soybean) [12], the bitter-tasting compounds of *Zanthoxylum bungeanum* Maxim. [13], the *kokumi* and bitter taste octadecadien-12-ynoic acids of chanterelles (*Cantharellus cibarius* Fr.) [14], and the kokumi-enhancing peptides of chicken protein hydrolysate [15].

The aim of this work is to characterize the major kokumi-enhancing compounds in green tea infusion. The kokumi-tasting fractions were separated based on sensory-guided separation and purification technologies. Taste dilution analysis (TDA) and taste dilution factor (TD-factor) were used to evaluate the *kokumi* intensity of each fraction. Ultra-high-performance liquid chromatography-tandem mass spectrometry (UHPLC-MS/MS) was used to identify the *kokumi* compounds. The impacts of other compounds commonly present in green tea infusion on *kokumi* taste were also investigated.

## 2. Results and Discussion

### 2.1. Separation of Peptides Fractions and Selection of Kokumi-Tasting Fractions

To separate the kokumi-peptides in green tea, a kokumi-flavored green tea sample (1:50 Huiming green tea) was fractioned by ultrafiltration with 3 kDa molecular weight cut-off and submitted to sensory evaluation. The sensory evaluation result showed that the ultrafiltration fractions with a molecular weight < 3 kDa were associated with *umami*, mellow, and thick taste, compared with the stronger astringent and bitter taste of the ultrafiltrated fractions with a molecular weight > 3 kDa. These results indicated that *kokumi* compounds remained in the fractions with a molecular weight < 3 kDa. To further evaluate the taste intensity of the ultrafiltration fractions, a taste dilution analysis (TDA) was conducted, and the taste dilution factor (TD-factor) of each fraction was calculated. The fraction with a higher TD-factor had a stronger *kokumi* intensity. The results showed that the fraction with a molecular weight < 3 kDa had a TD-factor of 9, while the fraction with a molecular weight > 3 kDa only had a TD-factor of 2 (Table 1). Hence, the fraction with a lower molecular weight (<3 kDa) contributed to the *kokumi* taste of green tea infusion, which was further separated by gel filtration chromatography.

**Figure 1:**
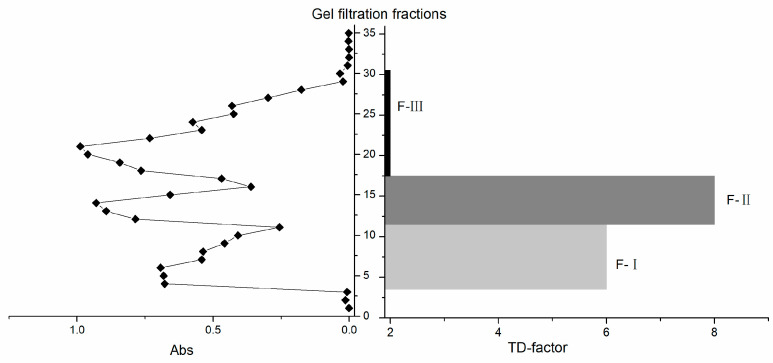
Gel chromatography and taste dilution (TD)-factors of kokumi-flavored green tea samples (Huiming green tea).

After gel filtration, 35 fractions (A1–A35) were collected and divided into three major fractions (F-I, F-II, and F-III) according to their elution sequence and absorbance at 220 nm [16] (Figure 1); specifically, F-I fraction consisted of A3–A10, F-II fraction consisted of A11–A16, and F-III fraction consisted of A17–A30. Fractions A1, A2, and A31–35 were not analyzed because of their extremely low absorbance values and they could not form a peak with the nearest fraction. Sensory evaluation results showed that the fraction F-I was characterized by astringent and slightly *umami* taste, and F-II was characterized by mellow and thick taste, while F-III was characterized by bitter and plain taste. To further evaluate the *kokumi* taste intensity of the gel filtration fractions, a taste dilution analysis (TDA) was conducted. The results showed that F-I had a TD-factor of 6, and F-II had a TD-factor of 8, while F-III only had a TD-factor of 2 (Table 1), indicating that F-I and F-II were closely associated with the *kokumi* taste of green tea infusion. Combining the results of sensory evaluation and TDA, it could be deduced that kokumi-tasting substances were mainly small molecular substances (<3 kDa) that were presented in F-I and F-II.

The well-known tea taste compounds, such as theanine (*umami*) and catechins (astringency), are small molecular substances (<1 kDa), and small molecular substances (<1 kDa) in certain mushrooms, such as *Agaricus bisporus* and chanterelles, have been confirmed to enhance the *kokumi* tastes [17,18]. Hence, it can be deduced that tea-flavored kokumi-tasting substances might be small molecular substances.

### 2.2. Analysis of Kokumi Peptides by UHPLC-MS

**Figure 2:**
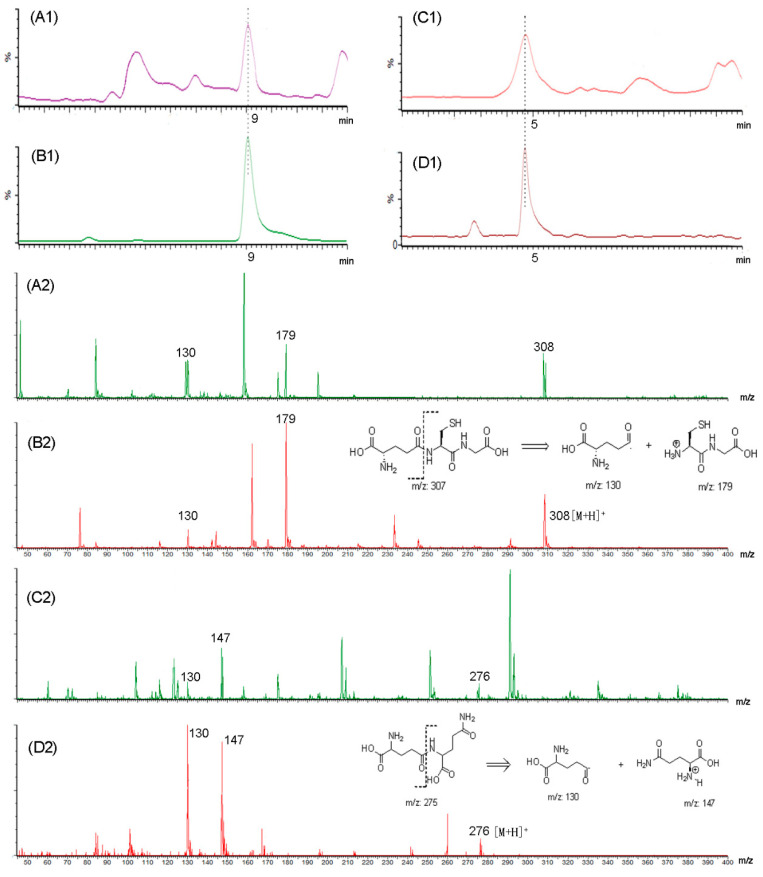
Ultra-high-performance liquid chromatography-tandem mass spectrometry (UHPLC) chromatography of (**A1**) F-I; (**B1**) GSH; (**C1**) F-II; (**D1**) γ-Glu-Gln; MS fragmentography of (**A2**) F-I; (**B2**) GSH; (**C2**) F-II; (**D2**) γ-Glu-Gln.

To understand the *kokumi* peptides in kokumi-tasting fractions, UHPLC-MS was applied for analysis. According to Toelstede et al. [19], 11 *kokumi* peptides were selected and their MRM channels are shown in Table S1 from Supplementary Materials. Among these MRM results, we chose graphs that were clear and use MS library and standards to confirm the compounds which were γ-Glu-Cys-Gly (GSH) and γ-Glu-Gln. Figure 2 shows the UHPLC chromatogram and MS information of F-I, γ-Glu-Cys-Gly (GSH), F-II, and γ-Glu-Gln. The compound in F-I was identified as GSH according to the retention time and MS information (Figure 2A1,A2), which was consistent with the retention time and MS data of GSH standard (Figure 2B1,B2). Accordingly, F-II shown in Figure 2C1,C2 was identified as γ-Glu-Gln based on the standard information (Figure 2D1,D2). The calibration curve of GSH was y = 2721.4x − 97.0 (r^2^ = 0.9922) and that of γ-Glu-Gln was y = 2269.2x + 287.9 (r^2^ = 0.9985).

To understand the distribution of GSH and γ-Glu-Gln in green tea samples with different *kokumi* intensities, 3 high-kokumi teas (HKs) and 3 low-kokumi teas (LKs) were selected for analysis under the concentration of 3 g/150 mL (tea-water ratio of 1:50). Table 2 showed the distribution of GSH content in high-kokumi-flavored green tea samples (HKs); this ranged from 8.50 μg/mL to 9.80 μg/mL, compared with 5.98 μg/mL to 6.96 μg/mL of low-kokumi-flavored green tea samples (LKs). The GSH contents in HKs were significantly higher than that in LKs. On the other hand, the γ-Glu-Gln content of high-kokumi-flavored green tea samples (HKs) ranged from 9.45 μg/mL to 12.50 μg/mL, compared with 5.49 μg/mL to 7.34 μg/mL of low-kokumi-flavored green tea samples (LKs). The γ-Glu-Gln contents of HKs were also significantly higher than those of LKs. Similarly, the total peptides contents of HKs (19.10–21.90 μg/mL) were significantly higher than that of LKs (12.28–14.30 μg/mL). These results suggest that the identified peptides may be the major *kokumi* peptides in green tea infusions.

### 2.3. The Chemicals Contributing to the Kokumi-Taste of Tea Infusions

**Figure 3:**
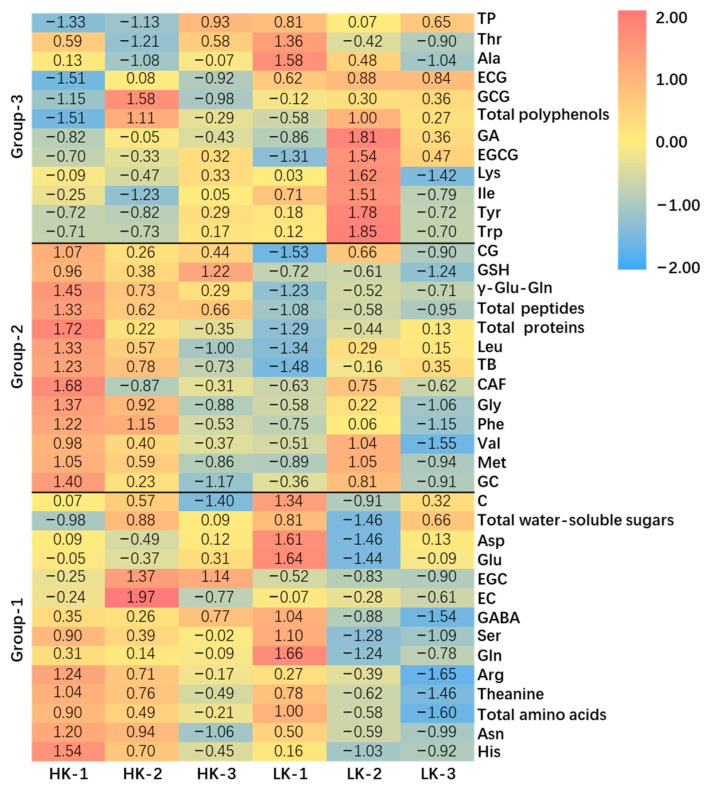
Heatmap of high-kokumi teas (HKs) and (low-kokumi teas) LKs.

To explore other potential contributors to the kokumi-taste of tea infusions, the following 39 chemical compositions within the above six tea samples (HKs and LKs, tea-water ratio of 1:50) were quantified, including 20 amino acids, 2 *kokumi* peptides, 8 catechins, 3 alkaloids, gallic acid (GA), total amino acids, total peptides (GSH and γ-Glu-Gln), total proteins, total polyphenols, and total water-soluble sugars. After row normalization, the 39 chemical compositions were automatically clustered into 3 groups by TBtools (https://github.com/CJ-Chen/TBtools accessed on 31 May 2022) software according to the distribution characteristics of substances among samples (Figure 3). Group-1 contained 14 compositions, including 9 amino acids (His, Asn, Theanine, Arg, Gln, Ser, GABA, Glu, and Asp), 3 non-gallated catechins (EC, EGC, and C), total amino acids, and total water-soluble sugars. These 14 compositions (Group-1) were mainly distributed in HK-1, HK-2, and LK-1, while rarely distributed in LK-2 and 3. Notably, the total amino acids contents of LK-1 were significantly higher than those of others (*p* < 0.05) (Table S2 from Supplementary Materials), because LK-1 represents Anji White tea, which is a special albino tea variety with high levels of amino acids [20]. However, the *kokumi* intensity of LK-1 was weaker than HKs, which may be due to the lower concentration of peptides in LK-1 (Table 2). Group-2 contained 13 compositions, including 5 amino acids (Met, Val, Phe, Gly, and Leu), 2 catechins (GC and CG), 2 *kokumi* peptides (GSH and γ-Glu-Gln), 2 alkaloids (CAF and TB), total proteins, and total peptides. These 13 compositions (Group-2) were mainly distributed in HK-1, HK-2, and LK-2, while rarely distributed in HK-3, LK-1, and LK-3. There were 3 compositions that showed higher concentrations in HKs, which were GSH, γ-Glu-Gln and total peptides. Group-3 contained 12 compositions, including 6 amino acids (Trp, Tyr, Ile, Lys, Ala, and Thr), 3 gallated catechins (EGCG, GCG, ECG), GA, TP, and total polyphenols. These 12 compositions (Group-3) were mainly distributed in LKs and HK-3, while rarely distributed in HK-1 and 2. In general, HKs contained more amino acids, *kokumi* peptides, and total proteins than LKs, while LKs contained higher concentrations of gallated catechins, GA and total polyphenols (Table S2). Gallated catechins were important contributors to the astringency of tea infusions [21].

The color scale bar for each compound indicates the abundance of the compound among six tea samples, and results in the same row are comparable. The normalization method was using Z-score, where Xzscore = (Xi − Xmean)/Xsd. The abbreviations and full names are as follows: Asp (aspartic acid), Glu (glutamic acid), Asn (asparaginase), Ser (serine), Gln (glutamine), His (histidine), Gly (glycine), Thr (threonine), Arg (arginine), Ala (alanine), GABA (γ-aminobutyric acid), Thea (theanine), Tyr (tyrosine), Val (valine), Met (methionine), Trp (tryptophan), Phe (phenylalanine), Ile (isoleucine), Leu (leucine), Lys (lysine), GC ((−)-gallocatechin), EGC ((−)-epigallocatechin), C ((+)-catechin), EC ((−)-epicatechin), EGCG ((−)-epigallocatechin gallate), GCG ((−)-gallocatechin gallate), ECG ((−)-epicatechin gallate), CG ((−)-catechin gallate), CAF (caffeine), TB (theobromine), TP (theophylline), GA (gallic acid), and total peptides (GSH and γ-Glu-Gln).

To further investigate the potential kokumi-taste compounds, principal component analysis (PCA), Pearson correlation, and sensory evaluation were employed. The data used were those in the heatmap and *kokumi* intensities in Table 2. According to Carr et al. [22], the cos (∠*kokumi*, *substance*) were calculated, and 20 substances (17 monomers and 3 mixtures) had cosines higher than 0.707, which indicated that they may have positive correlations with *kokumi*. The above 17 monomers’ taste profiles were evaluated by panelists under a 40 μg/mL concentration in Huiming green tea (1:150). Among them, only γ-Glu-Gln and GSH exhibited *kokumi* tastes, and theanine exhibited *umami* taste, while others showed bitter, astringent, and sweet tastes. In order to compare whether the correlation is significant, the Pearson correlation coefficient and *p*-value between *kokumi* and substances were calculated. The results showed that only γ-Glu-Gln, GSH, and their mixture (total peptides) had significant positive correlations with *kokumi* (*p* < 0.05) (Table 3 and Figure S1 from Supplementary Materials).

From the above results, γ-Glu-Gln and GSH were proved to be significantly correlated to the *kokumi* intensity of tea infusion. However, in other foods, amino acids were also positively contributed to *kokumi*. The concentrations of amino acids and *kokumi* peptides in fermented soybeans were increased by the addition of γ-Glutamyltranspeptidase (GGT) in the process of fermentation, which enhanced the *umami* and *kokumi* tastes [23]. A dynamic fermentation method of black tea could increase the contents of theaflavins, thearubigins, amino acids, and soluble sugars, leading to the enhanced *umami* tastes of black tea and ultimately resulting in superior sensory qualities [24]. These studies combined with the positive correlationship between *kokumi* and theanine in the above result made us wonder whether *umami* compound, theanine, has the potential to contribute to *kokumi*. Hence, theanine, the only amino acid, which showed an *umami* taste and had a positive correlation with *kokumi*, was further investigated in the next part.

### 2.4. Verification of the Effects of Identified Kokumi-Tasting Compounds

In order to verify the effects of identified kokumi-tasting compounds above, a sensory reconstruction experiment and quantitative description analysis (QDA) were carried out. Four samples were prepared for panelists to evaluate. The control sample was a basic tea infusion, which had the 5 basic but faint tastes (*kokumi*, *umami*, sweet, bitter, and astringent) of green tea with the same score of 0.50. Group-GSH was a 40 μg/mL GSH-Control sample solution, Group-theanine was an 80 μg/mL theanine-Control sample solution, and Group-mix was a GSH-theanine-Control sample solution (40 μg/mL GSH, 80 μg/mL theanine) (Table 4). In this experiment, γ-Glu-Gln was not involved because of the purification is difficult thus the price of standard is high. Since γ-Glu-Gln and GSH have the same taste profile (Table S3 from Supplementary Materials), we then only used GSH to verify peptides’ effect. 

**Figure 4:**
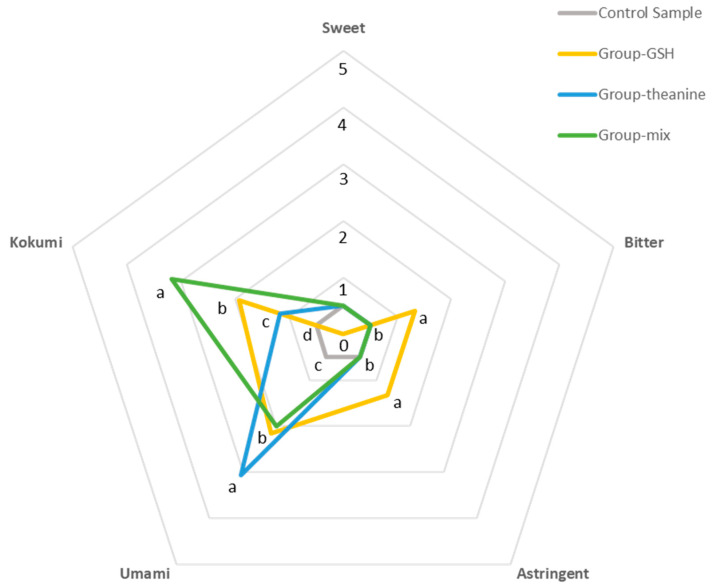
Spider plot of reconstituted samples.

Although these additives had no enhancing effect on the sweet taste, the intensities of other taste properties were enhanced to varying degrees, especially the *kokumi* taste. The intensities of *kokumi* taste were: Group-mix (3.17), Group-GSH (1.93), Group-theanine (1.17), and Control sample (0.50). Both theanine (Group-theanine) and GSH (Group-GSH) could significantly increase the *kokumi* intensities. Moreover, their mixture (Group-mix) exhibited a synergistic enhancing effect. For other taste properties, however, Group-mix showed no enhancing effects compared to other groups. Specifically, Group-GSH, Group-mix, and Group-theanine significantly enhanced the intensities of *umami*, among which theanine showed the most substantial enhancing effect. Besides, only Group-GSH significantly enhanced the astringent and bitter tastes. Therefore, GSH is confirmed to contribute to the *kokumi*, *umami*, astringent, and bitter tastes of tea infusions, while theanine mainly enhanced the *kokumi* and *umami* tastes of tea infusions. The intensity of *kokumi* was significantly increased when GSH and theanine were both presented in tea infusion (Figure 4 and Table S4 from Supplementary Materials).

Synergistic enhancing effects have been reported in numerous flavoring substances. Amino acids and organic acids can significantly enhance *umami* when co-existing in foods and beverages [25]. 5′-Mononucleotides and L-α-amino acids had a strong synergistic enhancing effect on *umami* taste [26]. Hence, it is reasonable to assume that amino acids are able to enhance specific tastes through synergistic effects with a variety of flavor compounds. Thus, we consider that theanine might enhance the *kokumi* flavor through the synergistic effect with the *kokumi* peptide GSH.

The preparations of solutions were shown in Table 4. The temperature of the solution at the time of sensory evaluation was about 55 °C. Different lowercase letters in the same taste attribute indicate significant differences between mean values (*p* < 0.05).

## 3. Materials and Methods

### 3.1. Chemicals and Materials

Sephadex G-15 was purchased from Shanghai Yuanye Bio-Technology Co., Ltd. (Shanghai, China); γ-Glu-Gln (≥98%) was purchased from GL Biochem, Ltd. (Shanghai, China). Glutathione (≥98%), NaOH (≥99%) and HPLC-grade formic acid were obtained from Aladdin Bio-Chem Technology Co., Ltd. (Shanghai, China); HPLC-grade methanol was obtained from Merck & Co., Inc. (Rahway, NJ, USA); Pellicon 2 Mini PLBC was purchased from EMD Millipore (Billerica, MA, USA). Individual free amino acids aspartic acid (Asp, ≥98%), glutamic acid (Glu, ≥98%), asparaginase (Asn, ≥98%), serine (Ser, ≥98%), glutamine (Gln, ≥98%), histidine (His, ≥98%), glycine (Gly, ≥98%), threonine (Thr, ≥98%), arginine (Arg, ≥98%), alanine (Ala, ≥98%), γ-aminobutyric acid (GABA, ≥98%), theanine (Thea, ≥98%), tyrosine (Tyr, ≥98%), valine (Val, ≥98%), methionine (Met, ≥98%), tryptophan (Trp, ≥98%), phenylalanine (Phe, ≥98%), isoleucine (Ile, ≥98%), leucine (Leu, ≥98%), and lysine (Lys, ≥98%) were purchased from Shanghai Yuanye Bio-Technology Co., Ltd. (Shanghai, China). The catechins, including (−)-gallocatechin (GC), (−)-epigallocatechin (EGC), (+)-catechin (C), (−)-epicatechin (EC), (−)-epigallocatechin gallate (EGCG), (−)-gallocatechin gallate (GCG), (−)-epicatechin gallate (ECG), and (−)-catechin gallate (CG), caffeine (CAF), theobromine (TB), theophylline (TP), and gallic acid (GA) were purchased from Aladdin (Shanghai, China). The ultra-pure water (>18 MΩ cm) was prepared by Milli-Q™ reference system (Merck Millipore, Milford, USA).

Six tea samples with different *kokumi* intensities were selected according to the results of sensory evaluations. Three tea samples with strong *kokumi* taste were Huiming green tea, Tangji green tea, and Dafo Longjing tea, which were termed HK-1, HK-2, and HK-3 in order. Three green tea samples with low *kokumi* intensity were Anji white tea, Yuezhou Longjing tea, and Xihu Longjing tea, which were termed LK-1, LK-2, and LK-3.

### 3.2. Ultrafiltration of Tea Infusions and Fraction Separation

According to the Chinese national standard GB/T 23776-2018 (methodology of sensory evaluation of tea), 3.00 g of tea sample was infused in 150 mL of 100 °C boiled water for 4 min. The tea infusion was filtered through a 0.45 μm microfiltration membrane and cooled to room temperature, followed by an ultrafiltration membrane separation of 3 kDa molecular weight cut-off (Pellicon 2 Mini PLBC). The ultrafiltration (<0.01 μm) was operated at room temperature (25 ± 3 °C) and under the pressure of 0.15 MPa. The filtrates with molecular weight less than 3 kDa or above 3 kDa were respectively concentrated by a rotary vacuum evaporator and freeze-dried for sensory evaluation and further separation.

Gel filtration chromatography, also known as gel exclusion chromatography or molecular sieve chromatography, was applied for further separation of the ultrafiltration mixture based on molecular size and polarity [27]. Three grams of the freeze-dried powder (molecular weight < 3 kDa) was dissolved in 30 mL of ultra-pure water, followed by a 0.45 μm membrane filtration. Then the filtrate was loaded to Sephadex G-15 column (2 × 60 cm) prerinsed by 1% formic acid aqueous solution (*w*/*w*). Formic acid aqueous solution (1%, *w*/*w*) was used for elution at a flow rate of 0.8 mL/min, and 35 fractions (A1–A35, 8 mL for each fraction) were collected. According to the absorbance at 220 nm of eluates [16], these 35 fractions were combined into three groups: F-I (A3–A10), F-II (A11–A16), and F-III (A17–A30). F-I, F-II, and F-III were respectively concentrated by a rotary vacuum evaporator and freeze-dried at −40 °C for 48 h. The freeze-dried powders were kept at −20 °C before sensory evaluation and chemical analysis.

### 3.3. Kokumi Taste Fractions Identified by Taste Dilution Analysis (TDA)

The taste profile of each fraction was described by sensory evaluation first. TDA procedure was modified and applied for the assessment of *kokumi* intensity [11]. Since the tastes of *kokumi* substances can be hardly sensed in water but clearly perceived in tea infusion (tea-water ratio of 1:150), tea infusion was used as the solvent for TDA. The powders achieved from ultrafiltration and gel filtration were dissolved in tea infusion at the concentration of 3 mg/mL and then stepwise 1 + 1-diluted with water. The serially diluted solutions were presented to eight panelists in order of decreased concentrations. The dilution fold at which no *kokumi* difference could be perceived between the diluted solution and tea infusion (tea-water ratio of 1:150) was defined as the taste dilution factor (TD-factor) of *kokumi*, and higher TD-factor represents stronger *kokumi* intensity. The temperature of the solution at the time of sensory evaluation was about 55 °C. Before and after each sample evaluation, water was offered to the panelists for palate cleansing. Each sample was evaluated twice by each panelist, and the mean value was presented.

### 3.4. Analysis of Peptides by UHPLC-MS/MS

The composition of peptides was analyzed using a UHPLC-MS/MS system (Waters, Milford, MA, USA). The peptides powders associated with *kokumi* taste were dissolved by ultrapure water at the concentration of 3 mg/mL. After filtration through a 0.22 μm membrane, 10 μL of the solution was injected into the UHPLC-MS system. The UHPLC conditions were: Waters Acquity UPLC HSST3 column (2.1 × 100 mm, 1.8 μm), column temperature 40 °C, injection volume 5 μL, mobile phase A = 0.1% formic acid + 99.9% water (*v*/*v*), mobile phase B = 0.1% formic acid + 99.9% methanol (*v*/*v*), flow rate 0.1 mL/min, linear gradient elution: starting with 100% phase A for the first 8 min, to 65% (v) A/35% (v) B at 10 min, to 1% (v) A/99% (v) B at 14 min, then keeping at 100% (v) A for 4 min re-equilibrium. An electrospray ionization (ESI) technique (positive ion mode) was employed for MS analysis. The ion source conditions were set as follows: capillary voltage 3 kV, cone voltage 30 V, extractor 3.0 V and RF lens 0.2 V, ion source temperature 150 °C, cone gas 50 L/h, desolvation gas at a flow rate of 600 L/h and temperature at 350 °C. Argon was used as the collision gas (collision energy of 16 Ev, flow rate 0.25 mL/min). LM resolution 1 and 2:15; HM resolution 1 and 2:15; ion energy 1 and 2:1. Full scan range of 45 to 600 atomic mass unit (amu) were recorded. Multiple reaction monitoring (MRM) method as shown in Table S1 from Supplementary Materials was used for determination of all the *kokumi* peptides. The acquired data were analyzed using MassLynx 4.0 software. The concentrations of GSH and γ-Glu-Gln were quantified by an external standard method. 

### 3.5. Spectrophotometric Analyses of Total Proteins, Amino Acids, Water-Soluble Sugars, and Polyphenols

The content of total proteins was determined by the Coomassie Brilliant Blue method; the content of total amino acids was determined by the ninhydrin method (GB/T 8313-2018); the content of total water-soluble sugars was determined by the anthrone-sulfuric acid method [28]; the content of total polyphenols was determined by the Folin-Ciocalteu method [29].

### 3.6. HPLC Analysis of Catechin Compounds and Amino Acids

The catechins were analyzed by the HPLC method (GB/T 8313-2018): Injection volume 10 μL, Agilent TC-C18 column (4.6 mm × 250 mm, 5 μm, Agilent Technologies, Santa Clara, CA, USA), column temperature 35 °C, mobile phase A = acetonitrile/acetic acid/EDTA-2Na/water (9:2:0.2:88.8, v), mobile phase B = acetonitrile/acetic acid/EDTA-2Na/water (80:2:0.2:17.8, v), linear gradient elution: starting with 100%A/0%B for the first 10 min, to 68%A/32% B at 25 min, keeping at 68%A/32% B for another 10 min, then keeping at 100%A/0%B at 55 min, flow rate 1 mL/min, detection wavelength 278 nm. 

The individual free amino acids were analyzed according to the reported method [30]. Prior to HPLC analysis, amino acids were derivatized by mixing 5 μL of sample with 500 μL of borate buffer (pH 10.2), 100 μL of ortho-phthalaldehyde (10 mg/mL), 100 μL of fluolenylmeghyl chloroformate (1.5 mg/mL), and 300 μL of deionized water. HPLC condition was: Zorbax Eclipse-AAA column (4.6 mm × 150 mm, 3.5 μm, Agilent Technologies Inc., Santa Clara, CA, USA), column temperature 40 °C, injection volume 10 μL. Mobile phase A = 40 mM Na_2_HPO_4_ (pH 7.8), mobile phase B = acetonitrile/methanol/water (45:5:10, *v/v/v*), linear gradient elution: linearly increasing from 95%A/5%B to 40%A/60%B during the first 18 min, then increasing to 0%A/100%B at 23 min, keeping at 95%A/5%B for 7 min re-equilibrium, flow rate 1.5 mL/min, fluorescent detector with the excitation wavelength of 340 nm and the emission wavelength of 450 nm.

### 3.7. Sensory Evaluation 

#### 3.7.1. Evaluation of *Kokumi* Intensity

Based on the reported method [31], the panelists rated the *kokumi* intensity of samples on a scale from 0 (not detectable) to 5 (intensively perceived). Green tea infusion was prepared by brewing tea sample (3.00 g) with 150 mL boiling water for 4 min, and then the green tea infusion was presented to panelists. Water was used as a palate cleanser between samples. The temperature of tea infusion at the time of evaluation was about 55 °C.

#### 3.7.2. Quantitative Descriptive Analysis (QDA) of Reconstituted Samples

QDA was used to evaluate the taste properties of tea samples according to the previous method [32]. Eight panelists (four males and four females, ages 20–35 years) were trained by the same taste references, including 1.5% glucose solution for sweetness, 0.2% theanine solution for *umami* taste, 0.1% quinine sulfate solution for bitterness, 0.75% EGCG solution for astringency, and a mixture of 0.8% glutathione and 0.4% theanine for tea-flavored *kokumi*. One gram of tea sample was brewed with 150 mL boiling water for 4 min, and the tea infusion (150 mL) with a certain additive agent labeled with a three-digit code was presented to each panelist in a randomized order for taste evaluation according to GB/T 23776-2018 (Methodology of sensory evaluation of tea). The panelists rated the intensity of taste attributes on a five-point scale (0: not detectable, 1: very weak, 2: a little weak, 3: neither weak nor strong, 4: a little strong, 5: very strong). The average score of each attribute was used for plotting the spider chart. Table 4 gives the information of reconstituted samples for sensory evaluation.

### 3.8. Statistical Analyses

Statistical analyses were conducted using XLSTAT statistical software (Version 2019.2.2; Addinsoft, New York, NY, USA). Fisher’s Least Significant Differences (LSD) were calculated at a 5% significance level to compare variable means. A probability level of 5% was considered significant. Pearson correlation analysis was conducted to investigate correlations with a confidence interval of 95%. The heatmap was illustrated by TBtools (https://github.com/CJ-Chen/TBtools accessed on 31 May 2022) software. Three repeats of each were used for the chemical analysis.

## 4. Conclusions

In this study, tea-flavored kokumi-tasting compounds in green tea infusions were investigated. GSH and γ-Glu-Gln were extracted and identified as major tea-flavored *kokumi* peptides. These two components had a remarkable effect on tea-flavored *kokumi* taste, despite their relatively low concentration in tea infusions. Sensory reconstitution evaluation results indicated that the addition of *kokumi* peptides significantly enhanced *kokumi* intensity of green tea infusion. Theanine could enhance the *kokumi* taste of tea infusion when coexisting with *kokumi* peptides. The *kokumi* substances isolated in our study can be used as flavor modifying agents in the production of tea beverages.

## Figures and Tables

**Table 1:** Sensory evaluation of ultrafiltration and gel filtration fractions of kokumi-flavored green tea samples (1:50 Huiming green tea).

Fraction	Taste Profile	TD-Factor
ultrafiltration fraction (<3 kDa)	*umami*, mellow and thick (*kokumi*), fresh, slightly astringent	9
ultrafiltration fraction (>3 kDa)	astringent, coarse, slight bitter, slightly burnt, slightly steamed and overcooked	2
F-I	astringent, slightly *umami*	6
F-II	mellow and thick (*kokumi*)	8
F-III	bitter, plain	2

F-I, F-II, and F-III represent the three fractions obtained by gel filtration of ultrafiltration fractions (<3 kDa), respectively. TD-factor is the dilution at which no discernable *kokumi* difference can be observed between diluted solution and tea infusion. The preparations of TDA solutions were using 1:150 Huiming green tea as a medium to make up 3 mg/mL solutions. The temperature of the solution at the time of sensory evaluation was about 55 °C.

**Table 2:** *Kokumi* intensity and content of *kokumi* peptides in different tea infusions.

	HK-1	HK-2	HK-3	LK-1	LK-2	LK-3
GSH (μg/mL)	9.40 ± 0.04 ^b^	8.50 ± 0.08 ^c^	9.80 ± 0.02 ^a^	6.79 ± 0.02 ^e^	6.96 ± 0.21 ^d^	5.98 ± 0.02 ^f^
γ-Glu-Gln (μg/mL)	12.50 ± 0.06 ^a^	10.60 ± 0.01 ^b^	9.45 ± 0.13 ^c^	5.49 ± 0. 02 ^f^	7.34 ± 0.22 ^d^	6.83 ± 0.07 ^e^
Total peptides (μg/mL)	21.90 ± 0.10 ^a^	19.10 ± 0.09 ^b^	19.25 ± 0.15 ^b^	12.28 ± 0.04 ^e^	14.30 ± 0.43 ^c^	12.81 ± 0.09 ^d^
*Kokumi* intensity	4.56 ± 0.42 ^a^	4.13 ± 0.44 ^b^	4.06 ± 0.82 ^b^	0.63 ± 0.52 ^d^	0.94 ± 0.56 ^cd^	1.13 ± 0.35 ^c^

Sample HKs (HK-1, HK-2, HK-3) represent high-kokumi-flavored green tea samples; sample LKs (LK-1, LK-2, LK-3) represent low-kokumi-flavored green tea samples. Total peptides = GSH + γ-Glu-Gln. The temperature of tea infusion at the time of sensory evaluation was about 55 °C. Different lowercase letters in the same row indicate significant differences between mean values (*p* < 0.05).

**Table 3:** Principal component analysis (PCA) loadings of variables and correlations between *kokumi* and substances.

Variable	PCA Loading		Cos (∠*Kokumi, Substance*)	Taste Profile (40 μg/mL)	Pearson Correlation between *kokumi* and Substance	
	F1	F2			*r*	*p*
His	0.933	0.317	0.925	bitter	0.670	0.146
Phe	0.913	−0.309	0.965	bitter	0.699	0.123
Arg	0.901	0.192	0.963	bitter	0.625	0.184
γ-Glu-Gln	0.896	−0.250	0.978	***kokumi***	**0.941**	**0.005**
Gly	0.882	−0.321	0.959	sweet	0.555	0.253
Total peptides	0.870	−0.158	0.993	/	0.975	0.001
Asn	0.821	0.264	0.931	bitter	0.399	0.433
*Kokumi* intensity	0.814	−0.050	1.000	/	1.000	/
Theanine	0.798	0.410	0.860	***umami***	**0.443**	**0.379**
Total proteins	0.737	−0.338	0.933	/	0.680	0.137
GSH	0.727	0.016	0.997	***kokumi***	**0.921**	**0.009**
Total amino acids	0.708	0.494	0.783	/	0.384	0.452
GC	0.673	−0.434	0.872	astringent	0.228	0.664
Val	0.658	−0.496	0.834	bitter	0.384	0.452
Ser	0.644	0.740	0.609	/	0.421	0.406
Met	0.632	−0.664	0.733	bitter	0.335	0.516
TB	0.622	−0.512	0.810	astringent	0.562	0.246
CG	0.622	−0.679	0.720	astringent	0.690	0.130
Leu	0.612	−0.610	0.750	bitter	0.428	0.397
CAF	0.512	−0.447	0.793	bitter	0.259	0.620
GABA	0.471	0.661	0.529	/	0.421	0.406
EC	0.447	0.001	0.998	astringent	0.315	0.543
EGC	0.437	0.140	0.932	astringent	0.756	0.082
Gln	0.293	0.913	0.246	/	0.066	0.901
Thr	0.097	0.629	0.091	/	−0.053	0.921
C	0.090	0.616	0.083	/	−0.286	0.583
Lys	0.004	−0.426	0.070	/	−0.116	0.827
Glu	−0.045	0.992	−0.107	/	−0.098	0.854
Asp	−0.057	0.974	−0.120	/	−0.145	0.784
Ala	−0.097	0.400	−0.296	/	−0.420	0.407
GCG	−0.174	−0.197	−0.613	/	−0.221	0.674
Total water-soluble sugars	−0.222	0.699	−0.361	/	−0.064	0.904
Ile	−0.346	−0.183	−0.854	/	−0.546	0.262
Total polyphenols	−0.415	−0.506	−0.585	/	−0.281	0.590
Trp	−0.426	−0.472	−0.623	/	−0.492	0.322
Tyr	−0.441	−0.423	−0.678	/	−0.488	0.326
EGCG	−0.458	−0.862	−0.414	/	−0.225	0.668
GA	−0.467	−0.839	−0.432	/	−0.450	0.371
ECG	−0.773	−0.106	−0.981	/	−0.875	0.022
TP	−0.872	0.320	−0.958	/	−0.611	0.197

The preparations of solutions were using 1:150 Huiming green tea as a medium to make up 40 μg/mL solutions. The temperature of the solution at the time of sensory evaluation was about 55 °C.

**Table 4:** The information of reconstituted samples for sensory evaluation.

Sample Name	Tea-Water Ratio (*w*/*v*)	Concentration in the Reconstituted Samples
Control sample	1:150	0
Group-GSH	1:150	GSH, 40 μg/mL
Group-theanine	1:150	Theanine, 80 μg/mL
Group-mix	1:150	GSH, 40 μg/mL; Theanine, 80 μg/mL

The preparations of solutions were using 1:150 Huiming green tea as a medium. The concentration of each additive agent was determined by pre-experiments (Table S5 from Supplementary Materials). The temperature of the solution at the time of sensory evaluation was about 55 °C.

## Data Availability

Not applicable.

## Supplementary Materials

The following supporting information can be downloaded at: https://www.mdpi.com/article/10.3390/molecules27175677/s1, Table S1. MRM channels of kokumi peptides; Table S2. Original data for heatmap of HKs and LKs; Table S3. Triangle test of GSH and γ-Glu-Gln; Table S4. Original data for spider plot; Table S5. Pre-experiment; Figure S1. PCA plot of variables.

## Sample Availability

Samples of the compounds mentioned above are available from the authors.
