# Supplementary material for: Sensory-Guided Identification and Characterization of Kokumi-Tasting Compounds in Green Tea (Camellia sinensis L.)
Source: Molecules. 2022 Sep 2;27(17):5677. doi: 10.3390/molecules27175677 (PMC9458127; doi:10.3390/molecules27175677)
Supplement: Supplementary file 1 [file molecules-27-05677-s001.zip › molecules-1822092-supplementary.pdf]

molecules

Supplementary Matterials

Sensory-Guided Identification and Characterization of Kokumi-Tasting Compounds in Green Tea (*Camellia sinensis* L.)

Jiachun Lu †, Yanyan Cao †, Yani Pan, Sifan Mei, Gang Zhang, Qiang Chu and Ping Chen *

Tea Research Institute, Zhejiang University, 866 Yuhangtang Road, Hangzhou 310058, China
* Correspondence: pingchen@zju.edu.cn; Tel.: +86-18857183162
† These authors contributed equally to this work.

Table S1. MRM channels of kokumi peptides.

Numbers	Peptides	Chemical formula	Parent ion m/z	Daughter ion m/z	MRM channel m/z
1	γ-Glu-Cys-Gly (GSH)	$C_{10}H_{17}N_3O_6S$	308	179	308 > 179
2	γ-Glu-Gln	$C_{10}H_{17}N_3O_6$	276	147	276 > 147
3	γ-Glu-Glu	$C_{10}H_{16}N_2O_7$	277	84	277 > 84
4	γ-Glu-Met	$C_{10}H_{18}N_2O_5S$	279	150	279 > 150
5	γ-Glu-His	$C_{11}H_{16}N_4O_5$	285	156	285 > 156
6	γ-Glu-Ala	$C_8H_{14}N_2O_5$	219	90	219 > 90
7	γ-Glu-Gly	$C_7H_{12}N_2O_5$	205	84	205 > 84
8	γ-Glu-Leu	$C_{11}H_{20}N_2O_5$	261	86	261 > 86
9	γ-Glu-Val	$C_{10}H_{18}N_2O_5$	247	72	247 > 72
10	γ-Glu-Phe	$C_{14}H_{18}N_2O_5$	311	136	311 > 136
11	γ-Glu-Tyr	$C_{14}H_{18}N_2O_6$	295	166	295 > 166

Table S2. Original data for the heatmap of HKs and LKs.

	HK-1	HK-2	HK-3	LK-1	LK-2	LK-3
GA	11.6 ± 0.64 e	15.32 ± 0.46 c	13.47 ± 0.25 d	11.44 ± 0.26 e	24.22 ± 0.42 a	17.27 ± 0.11 b
GC	30.23 ± 0.56 a	27.31 ± 0.41 c	23.79 ± 0.24 e	25.82 ± 0.13 d	28.74 ± 0.51 b	24.44 ± 0.27 e
EGC	292.96 ± 4.54 c	364.28 ± 4.33 a	354.09 ± 6.07 b	281.05 ± 3.55 d	267.16 ± 4.40 e	263.95 ± 3.23 e
C	20.38 ± 0.43 d	22.30 ± 0.39 b	14.63 ± 0.43 f	25.34 ± 0.30 a	16.55 ± 0.47 e	21.34 ± 0.12 c
EC	38.16 ± 1.57 b	78.14 ± 0.15 a	28.59 ± 0.23 c	41.28 ± 0.36 b	37.41 ± 0.36 b	31.60 ± 6.01 c
EGCG	566.55 ± 2.70 e	584.46 ± 1.89 d	616.21 ± 2.25 c	536.79 ± 3.49 f	675.88 ± 9.08 a	623.63 ± 9.22 b
GCG	86.46 ± 0.61 f	153.12 ± 0.55 a	90.59 ± 0.40 e	111.58 ± 0.27 d	121.75 ± 0.17 c	123.30 ± 0.27 b
ECG	111.19 ± 1.31 f	201.18 ± 0.33 d	144.50 ± 0.18 e	231.62 ± 0.38 c	246.38 ± 0.12 a	243.72 ± 0.39 b
CG	22.36 ± 0.33 a	18.75 ± 0.05 d	19.55 ± 0.34 c	10.72 ± 0.07 f	20.55 ± 0.26 b	13.52 ± 0.14 e
TB	20.39 ± 0.57 a	17.76 ± 0.48 b	8.99 ± 0.20 e	4.68 ± 0.03 f	12.32 ± 0.13 d	15.29 ± 0.17 c
TP	1.72 ± 0.22 c	1.87 ± 0.03 c	3.36 ± 0.19 a	3.27 ± 0.13 a	2.74 ± 0.14 b	3.16 ± 0.02 a
CAF	667.75 ± 9.37 a	441.19 ± 8.59 e	490.59 ± 7.20 c	462.80 ± 3.38 d	585.17 ± 4.17 b	463.52 ± 2.06 d
Total polyphenols	1179.89 ± 9.93 f	1449.53 ± 11.46 a	1305.42 ± 12.53 d	1275.64 ± 11.51 e	1438.63 ± 13.29 b	1362.77 ± 9.66 c
Asp	33.85 ± 0.36 b	29.78 ± 0.49 c	34.09 ± 0.34 b	44.63 ± 0.05 a	22.92 ± 0.23 d	34.15 ± 0.16 b
Glu	44.34 ± 0.56 c	39.56 ± 0.61 d	49.75 ± 0.43 b	69.68 ± 0.08 a	23.39 ± 0.29 e	43.76 ± 0.25 c
Asn	36.95 ± 0.13 a	34.12 ± 0.17 b	12.6 ± 0.12 e	29.39 ± 0.01 c	17.62 ± 0.08 d	3.36 ± 0.03 f
Ser	19.34 ± 0.89 a	17.38 ± 0.65 b	15.78 ± 0.46 c	20.1 ± 0.17 a	10.94 ± 0.31 d	11.7 ± 0.31 d
Gln	50.58 ± 0.23 b	45.69 ± 0.33 c	39.32 ± 0.23 d	88.89 ± 0.02 a	6.73 ± 0.15 f	19.81 ± 0.06 e
His	9.61 ± 0.18 a	7.13 ± 0.51 b	3.71 ± 0.36 d	5.51 ± 0.06 c	1.97 ± 0.24 e	2.31 ± 0.19 e
Gly	6.98 ± 0.05 a	5.98 ± 0.14 b	2.01 ± 0.1 e	2.68 ± 0.27 d	4.44 ± 0.07 c	1.62 ± 0.03 f
Thr	8.64 ± 0.37 b	5.23 ± 0.25 e	8.63 ± 0.18 b	10.1 ± 0.01 a	6.72 ± 0.12 c	5.81 ± 0.03 d
Arg	35.96 ± 0.71 a	30.71 ± 0.56 b	21.97 ± 0.39 d	26.38 ± 0.07 c	19.85 ± 0.26 e	7.34 ± 0.22 f
Ala	7.58 ± 0.11 c	4.16 ± 0.41 e	7.02 ± 0.29 d	11.73 ± 0.04 a	8.6 ± 0.19 b	4.25 ± 0.12 e
GABA	5.23 ± 0.14 c	5.08 ± 0.26 c	5.92 ± 0.18 b	6.35 ± 0.01 a	3.22 ± 0.12 d	2.15 ± 0.03 e
Theanine	275.98 ± 0.55 a	255.46 ± 0.44 c	163.74 ± 0.31 d	257.25 ± 0.04 b	154.35 ± 0.21 e	93.13 ± 0.12 f
Tyr	1.89 ± 0.17 c	1.45 ± 0.14 c	6.51 ± 0.1 b	6.04 ± 0.52 b	13.36 ± 0.07 a	1.91 ± 0.06 c
Val	10.08 ± 0.14 a	8.22 ± 0.28 b	5.77 ± 0.2 c	5.31 ± 0.02 d	10.27 ± 0.13 a	2.01 ± 0.06 e
Met	0.75 ± 0.01 a	0.58 ± 0.04 a	0.05 ± 0 b	0.04 ± 0 b	0.75 ± 0.26 a	0.02 ± 0 b
Trp	3.25 ± 0.06 c	3.17 ± 0.03 c	7.18 ± 0.02 b	6.96 ± 0.58 b	14.66 ± 0.01 a	3.31 ± 0.25 c
Phe	7.79 ± 0.61 a	7.63 ± 0.17 a	3.67 ± 0.12 c	3.16 ± 0.01 d	5.05 ± 0.08 b	2.21 ± 0.03 e
Ile	5.92 ± 0.12 d	3.25 ± 0.21 f	6.73 ± 0.15 c	8.53 ± 0.01 b	10.72 ± 0.1 a	4.45 ± 0.03 e
Leu	8.67 ± 0.17 a	6.94 ± 0.36 b	3.37 ± 0.25 d	2.61 ± 0.03 e	6.3 ± 0.17 c	5.98 ± 0.09 c
Lys	5.82 ± 0.43 bc	5.37 ± 0.42 c	6.31 ± 0.29 b	5.96 ± 0.04 b	7.83 ± 0.19 a	4.25 ± 0.12 d
Total Amino acids	579.5 ± 2.35 b	516.89 ± 2.89 c	409.67 ± 3.97 d	594.62 ± 4.23 a	353.33 ± 3.59 e	245.04 ± 4.71 f
GSH	9.40 ± 0.04 b	8.50 ± 0.08 c	9.80 ± 0.02 a	6.79 ± 0.02 e	6.96 ± 0.21 d	5.98 ± 0.02 f
γ-Glu-Gln	12.50 ± 0.06 a	10.60 ± 0.01 b	9.45 ± 0.13 c	5.49 ± 0. 02 f	7.34 ± 0.22 d	6.83 ± 0.07 e
Total peptides	21.90 ± 0.10 a	19.10 ± 0.09 b	19.25 ± 0.15 b	12.28 ± 0.04 e	14.30 ± 0.43 c	12.81 ± 0.09 d
Total water-soluble sugars	204.45 ± 9.62 c	261.37 ± 8.97 a	237.26 ± 7.63 b	259 ± 9.57 a	189.76 ± 5.43 d	254.65 ± 6.54 a
Total proteins	127.8 ± 2.47 a	114.59 ± 3.18 b	109.54 ± 6.76 bc	101.22 ± 2.78 c	108.68 ± 5.48 bc	113.78 ± 9.28 b

The abbreviations and full names are as follows: Asp (aspartic acid), Glu (glutamic acid), Asn (asparaginase), Ser (serine), Gln (glutamine), His (histidine), Gly (glycine), Thr (threonine), Arg (arginine), Ala (alanine), GABA (γ-aminobutyric acid), Thea (theanine), Tyr (tyrosine), Val (va-line), Met (methionine), Trp (tryptophan), Phe (phenylalanine), Ile (isoleucine), Leu (leucine), Lys (lysine), GC ((-)-gallocatechin), EGC ((-)-epigallocatechin), C ((+)-catechin), EC ((-)-epicatechin), EGCG ((-)-epigallocatechin gallate), GCG ((-)-gallocatechin gallate), ECG ((-)-epicatechin gallate), CG ((-)-catechin gallate), CAF (caffeine), TB (theobromine), TP (theophylline), GA (gallic acid), and total peptides (GSH and γ-Glu-Gln).Different lowercase letters in the same row indicate significant differences between mean values ($p < 0.05$)

Table S3. Triangle test of GSH and γ-Glu-Gln**.**

Panelist	Serving order	Corresponding number	Answer number	Result
1	A,A,B	208,527,690	208	Wrong
2	A,B,B	306,625,900	625	Wrong
3	A,B,B	554,434,435	435	Wrong
4	A,B,A	147,620,742	742	Wrong
5	B,A,A	870,234,751	234	Wrong
6	A,B,A	762,444,488	488	Wrong
7	B,B,A	919,280,160	919	Wrong
8	B,A,A	500,666,943	943	Wrong
9	B,A,B	766,327,448	766	Wrong
10	A,B,B	456,459,338	338	Wrong
11	B,B,A	857,936,260	936	Wrong
12	A,B,A	153,111,430	153	Wrong
13	B,A,A	894,531,335	531	Wrong
14	B,A,B	117,956,715	117	Wrong
15	B,A,A	445,489,127	489	Wrong
16	A,B,B	644,403,964	644	Right
17	A,A,B	580,783,740	740	Right
18	B,A,B	658,978,417	978	Right
19	B,B,A	948,827,905	905	Right
20	B,B,A	724,449,329	329	Right
21	A,A,B	908,150,272	272	Right
22	B,A,B	868,945,824	945	Right
23	A,B,A	691,770,530	770	Right
24	A,A,B	485,881,289	289	Right

Letter A represents a γ-Glu-Gln solution, letter B represents a GSH solution. The preparations of A, B solutions were using 1:150 Huiming green tea as a medium to make up 40 μg/mL solutions.The temperature of the solution at the time of evaluation was about 55 °C. In this experiment, only 9 panelists discriminated the difference (α > 0.2), which indicated that A and B had no sensory difference according to the Chinese national standard GB/T 12311-2012.

Table S4. Original data for the spider plot.

	Control Sample	Group-GSH	Group-theanine	Group-mix
Sweet	0.50 ± 0.00 ^{a}	0.00 ± 0.00 ^{a}	0.50 ± 0.00 ^{a}	0.50 ± 0.00 ^{a}
Bitter	0.50 ± 0.00 ^{b}	1.33 ± 0.29 ^{a}	0.50 ± 0.00 ^{b}	0.50 ± 0.00 ^{b}
Astringent	0.50 ± 0.00 ^{b}	1.33 ± 0.29 ^{a}	0.50 ± 0.00 ^{b}	0.50 ± 0.00 ^{b}
Umami	0.50 ± 0.00 ^{c}	2.17 ± 0.29 ^{b}	3.07 ± 0.12 ^{a}	2.00 ± 0.00 ^{b}
Kokumi	0.50 ± 0.00 ^{d}	1.93 ± 0.12 ^{b}	1.17 ± 0.29 ^{c}	3.17 ± 0.29 ^{a}

Different lowercase letters in the same row indicate significant differences between mean values ($p < 0.05$).

Table S5. Pre-experiment**.**

	Concentration (μg/mL)	Taste profile
GSH	20	slightly kokumi
	40	kokumi
	80	sour
	160	sour; astringent
Theanine	20	slightly umami
	40	slightly umami
	80	umami
	160	umami; slightly astringent

The preparations of solutions were using 1:150 Huiming green tea as medium.The temperature of the solution at the time of evaluation was about 55 °C.

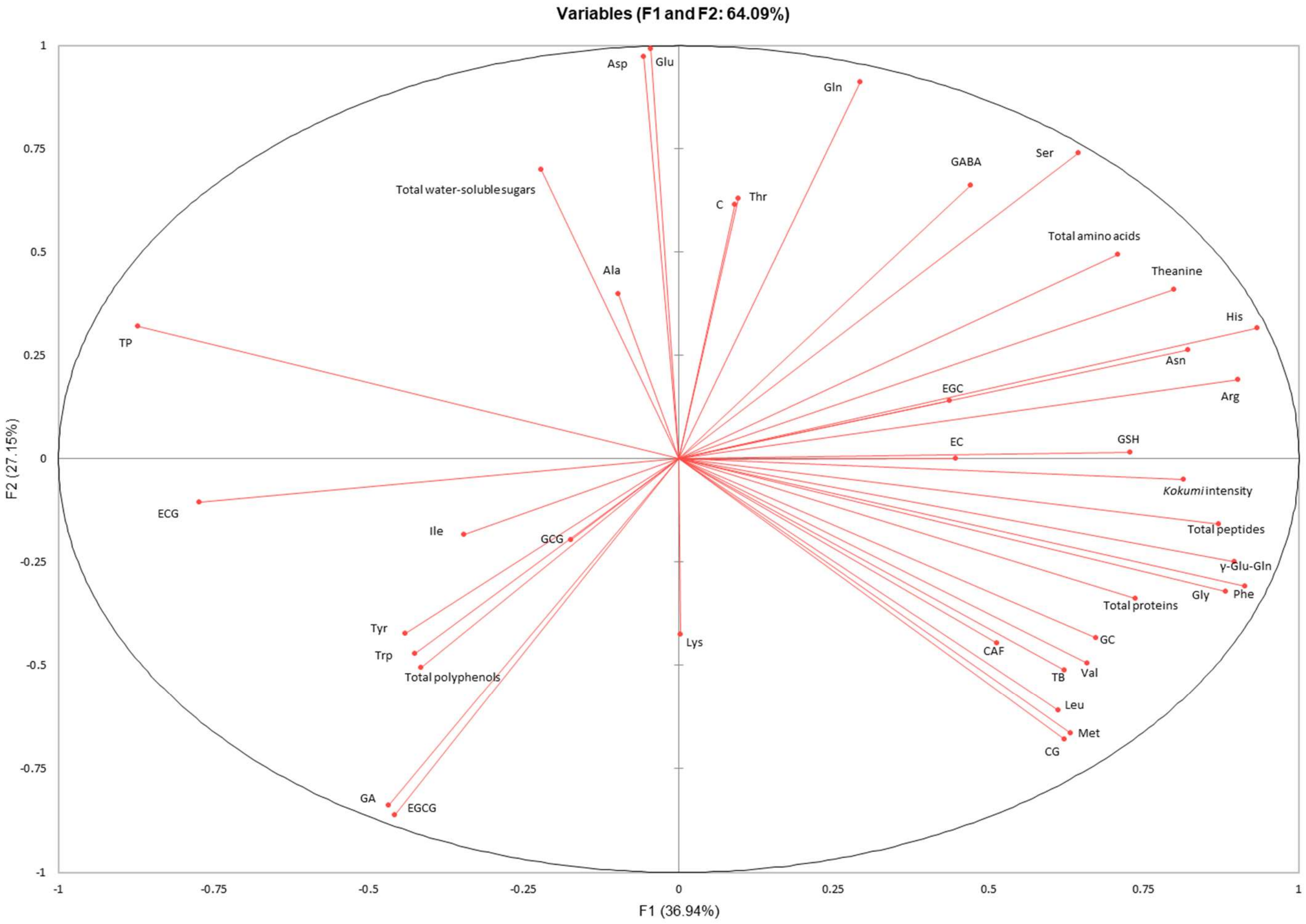

Figure S1. PCA plot of variables.
